# Fully Characterizing Lossy Catalytic Computation

**DOI:** 10.1007/s00453-026-01376-6

**Published:** 2026-05-20

**Authors:** Marten Folkertsma, Ian Mertz, Florian Speelman, Quinten Tupker

**Affiliations:** 1 CWI, Amsterdam, The Netherlands; 2 University of Warwick, England, UK; 3 University of Amsterdam, Amsterdam, The Netherlands; 4 QuSoft, Amsterdam, The Netherlands

## Abstract

A *catalytic machine* is a model of computation where a traditional space-bounded machine is augmented with an additional, significantly larger, “catalytic” tape, which, while being available as a work tape, has the caveat of being initialized with an arbitrary string, which must be preserved at the end of the computation. Despite this restriction, catalytic machines have been shown to have surprising additional power; a logspace machine with a polynomial length catalytic tape, known as *catalytic logspace* (${\textsf {CL}}$), can compute problems which are believed to be impossible for $\textsf {L}$. A fundamental question of the model is whether the catalytic condition, of leaving the catalytic tape in its exact original configuration, is robust to minor deviations. This study was initialized by Gupta et al. (2024), who defined *lossy catalytic logspace* (${\textsf {LCL}} [e]$) as a variant of ${\textsf {CL}}$ where we allow up to *e* errors when resetting the catalytic tape. They showed that ${\textsf {LCL}} [e] = {\textsf {CL}}$ for any $e = O(1)$, which remains the frontier of our understanding. In this work we completely characterize lossy catalytic space (${\textsf {LCSPACE}} [s,c,e]$) in terms of ordinary catalytic space (${\textsf {CSPACE}} [s,c]$). We show that

$$\begin{aligned} {\textsf {LCSPACE}} [s,c,e] = {\textsf {CSPACE}} [\Theta (s + e \log c), \Theta (c)] \end{aligned}$$

In other words, allowing *e* errors on a catalytic tape of length *c* is equivalent, up to a constant stretch, to an equivalent errorless catalytic machine with an additional $e \log c$ bits of ordinary working memory. As a consequence, we show that for any *e*, ${\textsf {LCL}} [e] = {\textsf {CL}}$ implies ${\textsf {SPACE}} [e \log n] \subseteq {\textsf {ZPP}}$, thus giving a barrier to any improvement beyond ${\textsf {LCL}} [O(1)] = {\textsf {CL}}$. We also extend all our results to every variant of catalytic space.

## Introduction

### Catalytic Computation

Within space-bounded computation, the *catalytic computing* framework, first introduced by Buhrman, Cleve, Koucký, Loff, and Speelman [2], models the question of whether or not full memory can be a computational resource. Their main object of study is a *catalytic logspace* (${\textsf {CL}}$) machine, in which a traditional logspace-bounded Turing machine is given access to a second work tape, polynomial in length, called the catalytic tape; while this tape is exponentially longer than the logspace work tape, it is already full with some string $\tau$ at the outset, and this string $\tau$ must be preserved by the overall computation.

Surprisingly, [2] show that ${\textsf {CL}}$ can be much more powerful than $\textsf {L}$, with the catalytic tape being at least as powerful a resource as non-determinism (${\textsf {NL}} \subseteq {\textsf {CL}}$), randomness (${\textsf {BPL}} \subseteq {\textsf {CL}}$), and more (${\textsf {TC}}^1\subseteq {\textsf {CL}}$). They also showed that its power is nevertheless limited and falls far short ${\textsf {PSPACE}}$, namely ${\textsf {CL}} \subseteq {\textsf {ZPP}}$. Later work showed improvements on both ends, with Agarwala and Mertz [1] showing that *bipartite maximum matching* is additionally in ${\textsf {CL}}$, and Cook et al. [10] showing that ${\textsf {CL}}$ reduces to the *lossy coding* problem, which is itself in ${\textsf {ZPP}}$.

This work spawned a long sequence of explorations of the power of catalytic space. Given the base model of ${\textsf {CL}}$ there are many possible variations and structural questions to be asked, such as the power of randomness [10, 15, 24], non-determinism [7, 24], non-uniformity [13, 14, 29, 31], and other variants [5, 21]. There have also been many works connecting the catalytic framework to broader questions in complexity theory, such as space-bounded derandomization [17, 27, 30], as well as adaptations of catalytic techniques to solve longstanding open questions such as compositional upper bounds for space [11, 12, 14]. This second line of work was recently used in a breakthrough result by Williams [32], showing that space is quadratically more powerful than time (see [25, 28] for surveys on the topic).

### Lossy Catalytic Computation

Besides these more standard structural questions, there are also catalytic variants which are more specific to the catalytic space restriction. In particular, Gupta et al. [22] initiated the study of *lossy* catalytic computing, wherein the catalytic tape need not be exactly reset to its initial configuration. This model, which we refer to as ${\textsf {LCSPACE}}$, essentially asks how robust the core definition of catalytic space is to seemingly small relaxations; for example, in the *quantum* setting [6], some computation error (albeit of a different form) is necessary for converting between different definitions based on allowed operations.

To begin, note that ${\textsf {CL}}$ with $e \le {{\,\textrm{poly}\,}}(n)$ errors trivially contains the class ${\textsf {SPACE}} [e]$ by simply erasing the first *e* bits of the catalytic tape and using them as free memory. Because we have not managed to prove that any space-bounded class beyond $\textsf {L}$ which is contained in ${\textsf {ZPP}}$, we should not expect to be able to prove ${\textsf {CL}}$ is the same as ${\textsf {CL}}$ with $e = \omega (\log n)$ errors. The question, then, is to understand where, in the range of $e = 0$ to $e = O(\log n)$, is the acceptable number of errors that ${\textsf {CL}}$ can provably tolerate.

As an initial answer to the previous question, [22] show that ${\textsf {CL}}$ gains no additional power from allowing any constant number of errors on the catalytic tape, i.e., ${\textsf {LCL}} [O(1)] = {\textsf {CL}}$. This remains the frontier of our knowledge, and Mertz [28] posed it as an open question to improve this result to any superconstant number of errors, or, alternatively, to provide evidence against being able to prove such a collapse.^1^ Recently, Cook et al. [10] showed that a different error-prone model, namely *randomized* ${\textsf {CL}}$, is no more powerful than the base ${\textsf {CL}}$ model, which was strengthened and expanded to *non-deterministic* ${\textsf {CL}}$ by Koucký et al. [24].

### Our Results

In this work we completely characterize lossy catalytic space in terms of ordinary catalytic space. Let ${\textsf {CSPACE}} [s,c]$ denote catalytic machines with free space *s* and catalytic space *c*, and let ${\textsf {LCSPACE}} [s,c,e]$ be the same with up to *e* errors allowed in resetting the catalytic tape. We show that these *e* errors are equivalent to an additional $e \log c$ free bits of memory, up to constant factor losses.

#### Theorem 1

Let $s:= s(n), c:= c(n), e:= e(n)$ be such that $e \le c^{1-\Omega (1)}$. Then

$$\begin{aligned} {\textsf {LCSPACE}} [O(s),O(c),e] = {\textsf {CSPACE}} [O(s + e \log c),O(c)] \end{aligned}$$

Besides characterizing ${\textsf {LCSPACE}} [s,c,e]$, the main takeaway of Theorem 1 is that allowing seemingly minor (superconstant) errors in the resetting condition can give an ${\textsf {LCSPACE}}$ machine surprising power. A concrete instantiation of this view is the following direct corollary.

#### Corollary 2

For any $$e:= e(n)$$,

$$\begin{aligned} {\textsf {LCL}} [e] = {\textsf {CL}} \quad \text{ implies } \quad {\textsf {SPACE}} [O(e \log n)] \subseteq {\textsf {ZPP}} \end{aligned}$$

If we revisit the assumption that we cannot hope to prove ${\textsf {SPACE}} [e \log n]$ is in ${\textsf {ZPP}}$ for any $e = \omega (1)$, then Corollary 2 implies the result of [22] is optimal with respect to *e*; any result of the form ${\textsf {LCL}} [\omega (1)] = {\textsf {CL}}$ is out of reach.

We also show that our proof extends to catalytic machines with additional power—usual examples include non-determinism, randomness, or non-uniformity—beyond errors; in fact, any “reasonable” catalytic setting is sufficient.

#### Theorem 3

Let  be any catalytic model such that , and let $s:= s(n), c:= c(n), e:= e(n)$ be such that $e \le c^{1-\Omega (1)}$. Then

This also gives a barrier to a more efficient removal of errors using additional resources, as Corollary 2 also applies to all other variants.

We briefly remark that the $e \le c^{1-\Omega (1)}$ restriction in all our results is only needed to get the constant stretch in the catalytic tape, and a different version holds in the general case:

#### Theorem 4

Let $$s:= s(n), c:= c(n), e:= e(n)$$. Then

$$\begin{aligned} {\textsf {LCSPACE}} [s,c,e] \subseteq {\textsf {CSPACE}} [s + O(e \log c),c] \subseteq {\textsf {LCSPACE}} [s,O(ec),e] \end{aligned}$$

While this version pays an additional factor of *e* in the catalytic space of the second inclusion, we also keep the number of errors fixed at exactly *e*; thus this result is somewhat incomparable to Theorem 1.

### Follow-up Work

In our original publication [20], we stated two open problems which have since been solved. First, Koucký et al. [24] showed a tight connection between randomized and deterministic catalytic space without errors, which, combined with Theorem 3, shows an equally tight and direct connection between lossy randomized catalytic space and ordinary (not-lossy, non-randomized) catalytic space. They also showed connections for non-deterministic and unbounded-error randomized catalytic space, but these results are not tight enough to utilize our work.

Second, in this version we strengthen Theorem 3 to work for any catalytic model, unlike the more specific cases of randomized and non-deterministic computation. In particular, this also includes non-uniform catalytic computing, i.e. catalytic branching programs, which, to the best of our knowledge, is the only other catalytic model with any substantive research.

### Open Problems

#### Errors in Expectation

A related question asked in [28] is whether or not ${\textsf {CL}}$ is equivalent to ${\textsf {CL}}$ with *O*(1) errors allowed *in expectation* over all starting catalytic tapes. This represents a different notion of distance between catalytic tapes, in opposition to Hamming distance, that may be more applicable to settings such as quantum computation. This question has received some attention in a related form by Bisoyi et al. [4], who introduce *almost* catalytic machines, which perfectly reset some catalytic tapes and are completely unrestricted on others.

However, no general results are known for expected errors—the results in [4] are very structured—and all techniques in our paper fail to restore the tape in pathological cases where a few starting tapes end up with potentially many errors. Furthermore, a barrier result was pointed out by an anonymous reviewer.^2^

#### Exact Simulation Space Requirements

In the current simulation of errors using clean space, we use $4e \log c$ clean space. By contrast, in our simulation of clean space using errors, we use only $(1+\epsilon )e$ more errors. If errors can be simulated in clean space $e \log c$ instead, then there is only very low overhead in switching between the two perspectives. This would tighten the correspondence between errors and space that we establish. However, since the distance between two codewords required to correct *e* errors is $2e + 1$, a different error correction code would be necessary to reach clean space $e \log c$.

## Preliminaries

We begin by defining catalytic machines as introduced by Buhrman et al. [2].

### Definition 1

(Catalytic space) A *catalytic Turing Machine* is a space-bounded Turing machine with two work tapes: 1) a read-write work tape of length *s*(*n*) which is initialized to $0^{s(n)}$, and 2) a read-write *catalytic tape* of length $c(n) \le 2^{s(n)}$ which is initialized to an arbitrary state $\tau \in \{0,1\}^{c(n)}$. On any input $x \in \{0,1\}^n$ and initial catalytic state $\tau$, a catalytic Turing machine has the property that at the end of the computation on input *x*, the catalytic tape will be in the initial state $\tau$.

In this work we focus on a relaxation of catalytic space by Gupta, Jain, and Sharma [22], where we are allowed to make some errors in resetting the catalytic tape.

### Definition 2

(Lossy catalytic space) A *lossy catalytic Turing Machine with* *e*(*n*) *errors* is a catalytic machine where at the end of the computation on any input $x \in \{0,1\}^n$ and initial catalytic state $\tau$, instead of requiring that the catalytic tape be in state $\tau$, the catalytic tape can be in any state $\tau '$ such that $\tau$ and $\tau '$ differ in at most *e*(*n*) locations.

Lastly we specify the basic complexity classes arising from our two catalytic definitions, as well as their specification to the “logspace” setting, where most research interest at the moment lies.

### Definition 3

We write

- ${\textsf {CSPACE}} [s,c]$: the class of languages which can be recognized by catalytic Turing Machines with work space $s:= s(n)$ and catalytic space $c:= c(n)$.
- ${\textsf {LCSPACE}} [s,c,e]$: the class of languages which can be recognized by lossy catalytic Turing Machines with work space $s:= s(n)$, catalytic space $c:= c(n)$, and $e:= e(n)$ errors.

We additionally write

- $${\textsf {CL}}:= {\textsf {CSPACE}} [O(\log n), {{\,\textrm{poly}\,}}n]$$
- $${\textsf {LCL}} [e]:= {\textsf {LCSPACE}} [O(\log n), {{\,\textrm{poly}\,}}n, e]$$

We note that throughout this paper we write $\mathcal {C}[O(f(n))]$ as a shorthand for $\bigcup _{c \in \mathbb {N}} \mathcal {C}[c \cdot f(n)]$ for complexity class $\mathcal {C}$ and function *f*(*n*).

## Main Theorem

In this section we will prove Theorem 1. We will do so via a simulation argument for each direction in turn.

### Simulating Errors with Space

First, we show that ${\textsf {LCSPACE}} [s,c,e] \subseteq {\textsf {CSPACE}} [O(s + e \log c),O(c)]$. In fact, we will not need any increase in the length of our catalytic tape.

#### Theorem 5

Let $$s:= s(n), c:= c(n), e:= e(n)$$. Then

$$\begin{aligned} {\textsf {LCSPACE}} [s,c,e] \subseteq {\textsf {CSPACE}} [s + O(e \log c),c] \end{aligned}$$

We note that this was also proven in [22] for the case of ${\textsf {LCL}} [O(1)]$, but we will pursue a different proof, based on error-correcting codes, which will allow us to generalize to other catalytic models in Section 4.

#### Proof

Let $M_e$ be an ${\textsf {LCSPACE}} [s,c,e]$ machine. We will devise a ${\textsf {CSPACE}} [s + O(e \log c), c]$ machine $M_0$ which simulates $M_e$. Note that in this section, we will not use our parameter restriction on *e*; this direction holds for every setting of *s*, *c*, and *e*. We will presume that $e \le \frac{c}{\log (c)}$, as the inclusion becomes trivial otherwise.

Our simulation will go via an error-correcting code. In particular we will use *BCH codes*^3^ (${\textsf {BCH}}$), named after Bose, Ray-Chaudhuri, and Hocquenghem [9, 23], which we define as per [16, 18]. We define the mapping ${\textsf {BCH}}$ and prove the following lemma in the appendix to the the full version of our paper.

#### Lemma 6

Let $q:= 2^{\lceil \log (c + e)\rceil }$. There exists a mapping ${\textsf {BCH}}: \mathbb {F}_q^q \rightarrow \mathbb {F}_q^q$ with the following operations:

- **Encoding:** ${\textsf {Enc}}_{{\textsf {BCH}}}$ takes as input a string *S* of length *c*, plus an additional $(2e + 1)\lceil \log (c + e) \rceil$ bits initialized in 0, and outputs a codeword $S_{enc}$:
  $$ S + [0]_{(2e + 1)\lceil \log (c + e) \rceil } \rightarrow _{{\textsf {Enc}}} S_{enc} $$
  Furthermore, all outputs $S_{enc}$ generated this way have minimum distance $\delta := 2e + 1$ from one another.
- **Decoding:** ${\textsf {Dec}}_{{\textsf {BCH}}}$ takes as input a string $S_{enc}'$ of length $c +(2e + 1)\log (c + e)$, with the promise that there exists a string *S* of length *c* such that ${\textsf {Enc}}_{{\textsf {BCH}}}(S + [0]_{2e \log (c + e)})$ differs from $S_{enc}'$ in at most $\delta /2 - 1 = e$ locations, and outputs this string *S*:
  $$ S_{enc}' \rightarrow _{{\textsf {Dec}}} S + [0]_{(2e+1)\log (c + e)} $$

Furthermore, both ${\textsf {Enc}}_{{\textsf {BCH}}}$ and ${\textsf {Dec}}_{{\textsf {BCH}}}$ are in place replacements of the input strings, they require at most an additional $O(e \log c)$ free space of memory.

We now move on to the simulation of our ${\textsf {LCSPACE}} [s,c,e]$ machine $M_e$. Our ${\textsf {CSPACE}} [s + O(e \log c),c]$ machine $M_0$ acts as follows:

1. **Initialization:** use the function ${\textsf {Enc}}_{{\textsf {BCH}}}$ to encode the initial state $\tau$ of the catalytic tape into a codeword, using $(2e+ 1)\lceil \log (c + e)\rceil$ additional bits from clean space,
  $$ \tau + [0]_{(2e + 1)\lceil \log (c + e)\rceil } \rightarrow _{{\textsf {Enc}}} \tau _{enc}. $$
2. **Simulation:** Run $M_e$ using clean space *s* and the first *c* bits of $\tau _{enc}$ as the catalytic tape.^4^ When $M_e$ finishes the calculation, we record the answer in a bit of the free work tape. The catalytic tape is, at this point, in a state $\tau _{enc}'$ which differs in at most *e* locations from $\tau _{enc}$.
3. **Cleanup:** use the function ${\textsf {Dec}}_{{\textsf {BCH}}}$ to detect and correct our resulting catalytic tape $\tau _{enc}'$:
  $$ \tau _{enc}' \rightarrow _{{\textsf {Dec}}} \tau + [0]_{(2e+1)\lceil \log (c + e)\rceil } $$
  Once we finish this process, we output our saved answer and halt.

The correctness of $M_0$ is clear, as it gives the same output as $M_e$. By our error guarantee on $M_e$ and the correctness of ${\textsf {Dec}}$, our catalytic tape is successfully reset to $\tau$. Our catalytic memory is *c* as before, while for our free work space we require *s* bits to simulate $M_e$, an additional $(2e + 1)\lceil \log (c+e)\rceil = (2+o(1))e \log c$ zero bits for our codewords, and $O(e \log c)$ space for ${\textsf {Enc}}_{{\textsf {BCH}}}$ and ${\textsf {Dec}}_{{\textsf {BCH}}}$, for $s + O(e \log c)$ space in total. $\square$

#### Note 3.1

There is an alternative proof of this point, one which gets better parameters and relies on an interesting characterization of space, namely the *reversibility* of space but which does not allow the strengthenings in Theorem 3. This proof is a simplification and extension of the one originally provided in [22], and we provide it in Appendix B for those interested.

### Simulating Space with Errors

We now show the other direction of Theorem 1, i.e. ${\textsf {CSPACE}} [s + e \log c,c] \subseteq {\textsf {LCSPACE}} [O(s),O(c),O(e)]$.

#### Theorem 7

Let $s {:}{=} s(n), c {:}{=} c(n), e := e(n), and~0< \Delta < log~c$ be such that . Then

$$\begin{aligned} {{\textsf {CSPACE}}}[s + e~\textrm{log}~c, c] \subseteq {{\textsf {LCSPACE}}}[s + \textrm{log}~c, (1 + o(1))c, (1 + \Delta )e] \end{aligned}$$

Since $s + log~c$ by the definition of a catalytic machine, this achieves the reverse direction of Theorem 1 with very small blowups in *s* and *c*, and for *e* bounded by a small polynomial in *c* we get a negligible error blowup as well. Furthermore, our proof is not limited to $e < c^{1/2}$; however, we will pay for larger values of *e* in the error blowup, and for  this factor becomes superconstant.

To understand our construction, we will first prove a version with looser space parameters. This result is incomparable to Theorem 7; although we lose a factor of *e* in our catalytic space, in exchange we have no restrictions on *e* and no loss in *e* either. In conjunction with Theorem 5, this also proves Theorem 4.

#### Theorem 8

Let $s:= s(n), c:= c(n), e:= e(n)$ be such that *c* is a power of 2. Then

$$\begin{aligned} {\textsf {CSPACE}} [s + e \log c,c] \subseteq {\textsf {LCSPACE}} [s,c + e(c + \log c),e] \end{aligned}$$

#### Proof

Let $M_0$ be a ${\textsf {CSPACE}} [s + e \log c, c]$ machine. We will devise a ${\textsf {LCSPACE}} [s,c + e(c + \log c),e]$ machine $M_e$ which simulates $M_0$.

Throughout this proof, we will associate $[2^k]$ with $\{0,1\}^k$ in the usual manner, i.e. subtracting 1 and taking the binary representation, and so we will use them interchangeably. Our workhorse is the following folklore^5^ construction:

#### Lemma 9

For every *k*, there exists a mapping ${\textsf {ind}}: \{0,1\}^{2^k} \rightarrow \{0,1\}^k$, computable in space $k+1$, such that the following holds: for any $\tau \in \{0,1\}^{2^k}$ and any $y \in \{0,1\}^k$,

$$\begin{aligned} {\textsf {ind}}(\tau \oplus \sigma _y) = {\textsf {ind}}(\tau ) \oplus y \end{aligned}$$

where $\sigma _y$ is the vector of length $2^k$ with a single 1 in position *y*.

**Fig. 1:**
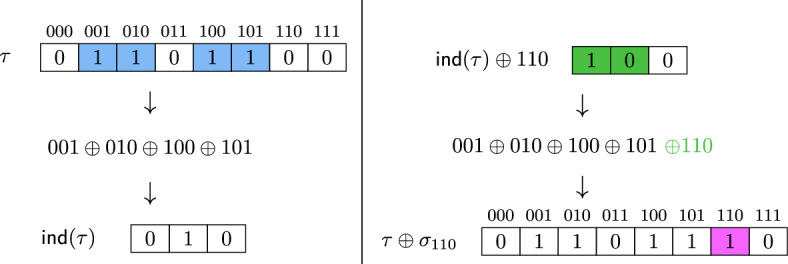
Example of our construction in Lemma 9 for $k=3$ and $\tau$ = 01101100: 1) calculating ${\textsf {ind}}(\tau )$ based on the positions of the 1s in $\tau$ (blue); 2) how flipping one bit of $\tau$ (purple) allows us to change ${\textsf {ind}}(\tau )$ (changes in green)

Intuitively, Lemma 9 gives us an easily computable mapping where any value of the *k*-bit output string can be given as output by flipping one bit of the $2^k$-bit input string (See Fig. 1).

#### Proof of Lemma 9

Let $\tau \in \{0,1\}^{2^k}$ be indexed by bitstrings $z \in \{0,1\}^k$. We will define our mapping ${\textsf {ind}}$ as the entrywise sum of all indices *z* where $\tau _z = 1$, i.e.

$$\begin{aligned} {\textsf {ind}}(\tau )_j = {\mathop {\bigoplus }\limits _{\begin{array}{c} {z \in \{0,1\}^k}\\ {z_j = 1} \end{array}}} \tau _z \end{aligned}$$

This is clearly computable in space $k+1$, as we need only store *z* and our current sum. Now note that for any *y*, flipping the entry $\tau _y$, i.e. $\tau \oplus \sigma _y$, flips every ${\textsf {ind}}(\tau )_j$ entry where $y_j = 1$ and leaves all other ${\textsf {ind}}(\tau )_j$ entry unchanged, which gives ${\textsf {ind}}(\tau ) \oplus y$ as claimed. $\square$

We now show how to simulate our ${\textsf {CSPACE}} [s + e \log c, c]$ machine $M_0$ by an ${\textsf {LCSPACE}} [s,c + e(c + \log c),e]$ machine $M_e$. First, let $\tau _0$ be the first *c* bits of catalytc memory, which we will set aside for simulating $M_0$. We will break the remaining $e \cdot (c + \log c)$ bits of our catalytic tape of $M_e$ into *e* blocks $B_1 \ldots B_e$ of size $2^k + k$ each, where $k = \log c$ (recall that *c* is a power of 2 by assumption). Within block $B_i$, let $\tau _i$ be the first $2^k$ bits and ${\textsf {mem}}_i$ be the remaining *k* bits. Our algorithm performs as follows:

1. **Initialization:** for each block $i \in [e]$, calculate $z_i = {\textsf {ind}}(\tau _i)$, set $y_i = {\textsf {mem}}_i \oplus z_i$, and update $\tau _i$ to
  $$\begin{aligned} \tau _i' \leftarrow \tau _i \oplus \sigma _{y_i} \end{aligned}$$
  By Lemma 9, after this step we have that
  $$\begin{aligned} {\textsf {ind}}(\tau _i') = z_i \oplus y_i = {\textsf {mem}}_i \qquad \forall i \in [e] \end{aligned}$$
  Finally zero out each block ${\textsf {mem}}_i$:
  $$ {\textsf {mem}}_i \leftarrow 0^k \qquad \forall i \in [e] $$
2. **Simulation:** run $M_0$ on catalytic tape $\tau _0$ with the work memory from $M_e$ plus $\{{\textsf {mem}}_i\}_{i \in [e]}$, for a total of
  $$\begin{aligned} s + ek = s + e \log c \end{aligned}$$
  free bits as necessary.
3. **Cleanup:** when we reach the end of $M_0$’s computation, record the answer on the free work tape and reset all the blocks ${\textsf {mem}}_i$ using ${\textsf {ind}}$:
  $$ {\textsf {mem}}_i \leftarrow {\textsf {ind}}(\tau _i') \qquad \forall i \in [e] $$
  We then return the saved answer and halt.

The correctness of $M_e$ is clear, as we output the same value as $M_0$. We require $c + e(c + \log c)$ catalytic bits plus *s* free bits for our simulation, while ${\textsf {ind}}$ can be computed in space $k = \log c \le s$ by assumption; thus all our memory is as claimed.

We also claim that our lossy catalytic condition is satisfied. Each $\tau _i'$ is at most one error away from $\tau _i$ in the initialization phase, and is never altered again, giving a total of *e* errors. By the property of $M_0$, there are no errors made to $\tau _0$ during the simulation step. Lastly, by the property that ${\textsf {ind}}(\tau _i') = {\textsf {mem}}_i$, the cleanup step exactly resets the blocks ${\textsf {mem}}_i$, meaning no further errors are introduced to the catalytic tape. $\square$

We now return to Theorem 7, which requires only a small modification of the above proof, namely to break the the catalytic tape into more, smaller blocks, which reduces its required length at the cost of a few extra errors. This modification works because the number of pure bits represented is logarithmic in the length of the block, and so making the blocks smaller barely affects the number of bits represented; for example, *c*/2 bits in a block still lets you represent $\log (c) - 1$ bits, so half the size only loses one bit per block.

#### Proof of Theorem 7

Let $M_0$ be a $\textrm{CSPACE}[s + e \log c, c]$ machine. We will devise a $\textrm{LCSPACE}[s,(1+o(1))c,(1+\Delta )e]$ machine $M_e$ which simulates $M_0$, where $\Delta$ satisfies $e \ll (1 + \Delta ) \cdot c^{1-1/(1+\Delta )})$.

We will have the same approach as Theorem 8, but now we use $(1+\epsilon ) e$ blocks of length $2^{k'} + k'$, where

$$\begin{aligned} k' = \bigg \lceil \frac{\log c}{1+\epsilon } \bigg \rceil \end{aligned}$$

Clearly we make at most $(1+\epsilon ) e$ errors by the above analysis, while our free space is

$$\begin{aligned} s+ (1 + \Delta )e \cdot k' = s + (1 + \Delta )e \cdot \bigg \lceil \frac{log c}{1+\Delta }\bigg \rceil \ge s + e log c \end{aligned}$$

Finally we analyze our catalytic memory. Our $\tau _i'$ blocks give us a total usage of

$$\begin{aligned} (1 + \Delta )e \cdot 2^{k'} = (1 + \Delta )e \cdot 2^{\lceil log c/(1+\Delta )\rceil } \le (1 + \Delta )e \cdot \left( 2 c^{1/(1+\Delta )}\right) \ll c \end{aligned}$$

where the last line follows by our bound on $e = o(c^{\epsilon /(1+\epsilon )})$.

We will use our memory $\{\tau _i\}_{i\in [(1+\Delta ) log c]}$ for the simulation of $M_0$, plus enough extra catalytic memory $\tau _0$ needed to reach *c* total bits. Since $M_0$ exactly resets its catalytic tape this introduces no new errors, and together with the ${\textsf {mem}}_i$ blocks, this gives us a total catalytic memory of

$$\begin{aligned} c + (1+\epsilon )e \cdot k' = c + e \log c + O(1) = (1+o(1))c \end{aligned}$$

which completes the proof. $\square$

## Further Consequences

With this, we have concluded our main theorem and proof. We now move to corollaries and extensions.

### Lossy Catalytic Logspace with Superconstant Errors

As stated in the introduction, it immediately follows from Theorem 1 that proving ${\textsf {LCL}} [e] = {\textsf {CL}}$ is likely difficult, if not false, for superconstant values of *e*.

#### Proof of Corollary 2

This follows immediately from the fact that

$$\begin{aligned} {\textsf {LCSPACE}} [O(\log n),{{\,\textrm{poly}\,}}n,e]&= {\textsf {CSPACE}} [O(\log n + e \log ({{\,\textrm{poly}\,}}n)), {{\,\textrm{poly}\,}}n] \\&= {\textsf {CSPACE}} [O(e \log n), {{\,\textrm{poly}\,}}n] \\&\supseteq {\textsf {SPACE}} [O(e \log n)] \end{aligned}$$

combined with the fact that ${\textsf {CL}} \subseteq {\textsf {ZPP}}$ by [2]. $\square$

### Lossy Catalytic Space with Other Resources

As mentioned in Section 1, there are many extensions of the base catalytic model besides ${\textsf {LCSPACE}}$, such as randomized, non-deterministic, and non-uniform ${\textsf {CSPACE}}$. So far, however, there has been little discussion of classes where more than one such external resource has been utilized. In this section we observe that our proof of Theorem 1 carries through no matter what base catalytic model we are using, even if we are granted additional resources which the errors can depend on.

#### Proof sketch of Theorem 3

As earlier, we need to show both directions. We will prove the same two equivalences as in Theorems 5 and 7, namely

In both cases we only need check two computations. First we simulate our machine $M_0$/$M_e$ via a machine $M_e$/$M_0$ (respectively) which is given direct access to the appropriate amount of work and catalytic memory; by definition this can be done irrespective of . Second is our two mappings needed to reset the catalytic tape at the end; since ${\textsf {SPACE}} [s]$ can implement both our BCH codes and the mapping ${\textsf {ind}}$, by assumption  can do so as well. $\square$

## Data Availability

No datasets were generated or analysed during the current study.

## Simulating Errors with Space Via Reversibility

In this section we give an alternate proof of simulating ${\textsf {LCSPACE}}$ via ${\textsf {CSPACE}}$, with sharper parameters than those in Theorem 5.

### Theorem 10

Let $$s:= s(n), c:= c(n), e:= e(n)$$. Then

$$\begin{aligned} {\textsf {LCSPACE}} [s,c,e] \subseteq {\textsf {CSPACE}} [s + (e+1) \log c,c] . \end{aligned}$$

For this proof, we need to invoke a property of space-bounded machines known as *reversibility*, which we define now.

### Definition 4

A Turing machine *M* is (strongly) *reversible* if the following conditions hold:

1. For any input *x*, every node *v* in its configuration graph $G_x$ has both in-degree and out-degree at most one. Let $for_x(v)$ indicate the unique node with a directed edge $(v,for_x(v))$, and let $back_x(v)$ indicate the unique node with a directed edge $(back_x(v),v)$.
2. There exist machines $M_{\rightarrow }$ and $M_{\leftarrow }$ such that for every node *v* in the configuration graph of *M*, $M_{\rightarrow }(x)$ sends *v* to $for_x(v)$ and $M_{\leftarrow }$ sends *v* to $back_x(v)$.

A classical result of Lange, McKenzie, and Tapp [26] shows that every ${\textsf {SPACE}} [s]$ machine can be made reversible with no additional space. Dulek [19] showed the same result for catalytic machines, while Gupta et al. [22] extended this to catalytic machines with error; both of the latter results use a very similar Eulerian tour argument to [26].

### Lemma 11

Let *M* be a ${\textsf {CSPACE}} [s,c]$ (resp. ${\textsf {LCSPACE}} [s,c,e]$) machine recognizing language *L*. Then there exists a reversible ${\textsf {CSPACE}} [s,c]$ (resp. ${\textsf {LCSPACE}} [s,c,e]$) machine $M'$ which recognizes *L*.

In light of Lemma 11, it seems that there is nothing interesting to be said about ${\textsf {LCSPACE}}$; after all, can we not simply reverse our machine to the starting point, wherein there are no errors on the catalytic tape? While this is technically true, there may be many different starting configurations which reach the same halting state $(\tau ,v)$. All such start states can and will be reached by running $M_{\leftarrow }$ from $(\tau ,v)$ for long enough, but without knowledge of which particular start state we began with, this naïve reversing procedure cannot reset our catalytic tape free of error.

Nevertheless, a small tweak on this idea, using our additional $(e+1) \log c$ bits, immediately works.

### Proof of Theorem 10

Let $M_e$ be a ${\textsf {LCSPACE}} [s,c,e]$ machine, and by Lemma 11 we will assume $M_e$ is reversible. We will devise a ${\textsf {CSPACE}} [s + (e+1) \log c, c]$ machine $M_0$ which simulates $M_e$.

We will assume without loss of generality that all start and end states of a catalytic machine *M* are distinguished; for example, we traditionally assume any state with an all-zeroes work tape is a start state. We write $\textsf {start}(\tau )$ to indicate the unique start state of *M* with initial catalytic tape $\tau$, while we write $\textsf {end}_x(\tau )$ to indicate the unique end state reached by *M* from initial state $\textsf {start}(\tau )$ on input *x*.

Now let $S_{x,(\tau ,v)}:= \{\textsf {start}(\tau _i)\}_{i}$ be the set of start states such that $\textsf {end}_x(\tau _i) = (\tau ,v)$. Since $M_e$ is an ${\textsf {LCSPACE}} [s+\log c,c,e]$ machine, each $\tau _i$ can differ from $\tau$ in at most *e* locations, and thus

$$\begin{aligned} |S_{x,(\tau ,v)}| \le {c \atopwithdelims ()\le e} \le \frac{c^{e+1}}{2} \end{aligned}$$

Our machine $M_0$ thus works as follows:

1. initialize a counter $num\_start$ with $\log {c \atopwithdelims ()\le e}$ bits to 0
2. simulate $M_e$ using $s \log c$ work bits and *c* catalytic bits, incrementing $num\_start$ each time we encounter a start state $\textsf {start}(\tau _i)$, until we reach an end state $(\tau ,v)$
3. record our answer and run $M_e$ in reverse, decrementing $num\_start$ each time we encounter a start state
4. halt when we reach a start state and $num\_start = 0$, and return our recorded answer

Clearly our algorithm outputs the correct answer, resets the catalytic tape exactly, and uses at most $s + 1 + (e + 1)\log c - 1$ bits of work memory plus *c* bits of catalytic memory. $\square$

We defer this discussion to the appendix for two reasons. First, the error-correcting approach more directly applies in both directions of Theorem 1; while Lemma 11 connects to Theorem 7 and can be applied before the final resetting step, this does not provide any qualitative or quantitative gain. Second, the reliance on reversibility makes the proof unsuitable to our later generalizations from Section 4.2; in particular, randomized, non-deterministic, and non-uniform catalytic computations are only reversible in a limited sense, one which rules out using Lemma 11.

## Space Efficient Linear Algebra on Finite Fields

### The Space Complexity of Solving Linear Systems

We prove the space efficiency of various common arithmetic and linear algebra operations necessary in order to encode and decode BCH codes. First, we introduce the concept of well-endowed rings [3]. This allows us to use earlier results to argue about the efficiency of various operations on rings without having to reprove those ourselves. The fields of interests are fields of the form $GF(p^{r_n})$ for a fixed prime *p* and a sequence $r_n$. Our results will apply to a field whose size increases asymptotically. Hence the uniformity of the calculations involved is important. But we assume that *p* is fixed for all fields.

All these results are expressed in their asymptotic complexity in terms of the size of the ring or a length function, which may be seen as a measure of the number of bits necessary to write down a value in a ring.

#### Definition 5

A length function $\rho$ for a ring *R* is a function satisfying that for any $x, y \in R$

1. $$\rho (x + y) \le \max \{\rho (x), \rho (y)\} + O(1)$$
2. $$\rho (xy) \le \rho (x) + \rho (y) + O(\log \max \{\rho (x), \rho (y)\})$$

An example is the number of bits of an integer.

From here we can define well-endowed rings as those with efficient implementations of addition, negation and multiplication.

#### Definition 6

A ring *R* with length function $\rho$ is *well endowed* if there is a succinct uniform representation in which it has efficient implementations of addition, negation and multiplication. Addition and negation are considered efficient if they can be implemented in (logspace uniform) $\textsf{NC}^0$ and multiplication is considered efficient if it can be implemented in (logspace uniform) $\textsf{NC}^1$. The parameter for $\textsf{NC}^1$ functions is always the length function of the ring.

We now argue that basic arithmetic can be done space efficiently. This is done in the following steps. First, we argue that the polynomial ring $GF(\xi )$ is well endowed and therefore we can perform polynomial addition, negation and multiplication efficiently. Then we argue that we can use this to compute the remainder of polynomial division efficiently. This allows to find an irreducible polynomial to represent the field $GF(p^{r_n})$ in order to perform addition, negation and multiplication efficiently. We finally show that we can evaluate multiplicative inverses inefficiently and use this to do division. With inefficiently we mean in space $O(\log |F|)$ for a field *F* whereas addition, negation and multiplication can be performed in space $O(\log \log |F|)$.

#### Lemma 12

For fixed *p*, the ring $GF(p)[\xi ]$ is well endowed.

#### Proof

We argue that finite fields are well-endowed rings. First observe that *GF*(*p*) for a fixed *p* is always well endowed since the size of the ring is independent of *n* so addition, negation and multiplication can be performed in $\textsf{NC}^0$. By Proposition 3.9 from [3] this means that polynomials over $GF(p)[\xi ]$ are also well endowed. The length function here is *O*(*d*) for a polynomial of degree *d*. Since they are well endowed, one can perform addition, negation and multiplication in space $O(\log d)$ for polynomials. $\square$

We use this to compute the remainder.

#### Lemma 13

Given polynomials $N(\xi )$ and $D(\xi )$ in $GF(2)[\xi ]$ of degree at most $r_n$, it is possible to compute the remainder $R(\xi )$ using an additional $r_n + O(\log r_n)$ space. If we can overwrite $N(\xi )$ in place, the additional space necessary is $\lceil \log r_n \rceil + O(1)$.

#### Proof

Suppose that $\chi \in GF(p)$ is the leading coefficient of *D*(*x*), we can compute and store $\chi ^{-1}$ in constant space since 2 is constant. We perform a kind of Gaussian elimination to compute the remainder:

1. If the degree of $D(\xi )$ exceeds the degree of $N(\xi )$ then return $N(\xi )$.
2. Let $\psi$ be the leading coefficient of $N(\xi )$. Compute $N(\xi ) + -\psi \chi ^{-1} D(\xi )$ overwriting $N(\xi )$ in the process. Since we use fixed field *GF*(2), this can be done in constant depth. Repeating this for each coefficient uses $O(\log r_n)$ space for a counter. We use *O*(1) to store $\psi$ and the coefficient of $N(\xi )$ during the computation. We then compute $N(\xi ) + - \psi ^{-1} D(\xi )$ coefficient by coefficient.
3. return to step 1.

Overall, we manage to compute the remainder in space $r_n + O(1)$ by copying the final remainder to a new part of the space and then updating it in place during every iteration. If we can overwrite $N(\xi )$ in the process, then the additional space required is only $r_n$ to keep track of a counter. $\square$

We can now search for irreducible polynomials.

#### Lemma 14

Given a sequence of positive integers $r_n$ and a constant prime *p*, it is possible to find a degree $r_n$ irreducible polynomial in $GF(p)[\xi ]$ in space $3 r_n + O(\log r_n)$.

#### Proof

It costs *d* space to store a polynomial over *GF*(*p*) of degree at most *d*. Therefore, one can iterate over all such polynomials. If we store two such polynomials and iterate over all pairs, the first can be a candidate irreducible polynomial, while the second can be a candidate factor of the first polynomial. By using Lemma 13 to test whether or not the candidate irreducible polynomial is divisible by the candidate factor in additional space $r_n + O(\log r_n)$, we can test if the candidate irreducible polynomial is irreducible. This uses an additional *d* space to store the remainder as it is calculated. Irreducible polynomials are guaranteed to exist, so we must find one eventually. $\square$

Together these results allow us to do division in $GF(q^r)$.

#### Lemma 15

Given a sequence of finite fields $F_n = GF(p^{r_n})$ for a constant prime *p*, it is possible to compute the multiplicative inverse of an element $x \in GF(p^{r_n})$ in additional space $4 r_n + O(\log r_n)$ counting the space needed to store the irreducible polynomial.

#### Proof

If $$x = 0$$ then there is no multiplicative inverse. Otherwise, try multiplying *x* by every other possible *y* and taking the remainder using Lemma 13 in place in space $$4 r_n )$$ until one finds a *y* such that $$xy = 1$$. It takes $$r_n $$ space to iterate over all possible *y*. For every *x*, *y*, we use another register to store *xy*. Storing *xy* needs an additional $$2 r_n $$ space, since we first need to compute the product as a product of polynomials and only take the remainder later. Computing *xy* uses an additional space $$O(\log r_n)$$ since the ring of polynomials over *GF*(*p*) is well endowed. Finally, we can use Lemma 13 to take the remainder in place in only $$\lceil \log r_n \rceil + O(1)$$. $$\square $$

We can now finally solve linear systems.

#### Lemma 16

Given a sequence of finite fields $F_n = GF(p^{r_n})$ for a constant prime *p*, it is possible to solve a linear system of $t_n$ equations and $t_n$ unknowns in $2t_n r_n + 5 r_n + O(\log ^2 r_n + \log t_n)$ space if $t_n = O(|F_n|)$ and we count the space used to store the irreducible polynomial for our representation of $GF(p^{r_n})$.

#### Proof

By Proposition 4.2 from [3] it is possible to compute the determinant over a well endowed ring in $$\textsf{NC}^2$$. By Lemma 12, we can perform this computation by treating elements of $$F_n$$ as polynomials over *GF*(*p*) first. By Theorem 4 from [8] this can be done in space $$O(\log ^2 \log |F_n|)$$. The cost of storing the polynomial representing the remainder is $$t_n r_n $$ since each entry of $$F_n$$ uses $$r_n $$ bits and the determinant is a sum of the product of at most $$t_n$$ elements. Then we can find an irreducible polynomial (or preferably access one that has been precomputed) in space $$3r_n + O(\log r_n)$$ by Lemma 14 and take the remainder in place with using additional space $$\lceil \log t_n r_n \rceil $$ by Lemma 13. This allows us to calculate determinants of $$t_n \times t_n$$ matrices over $$F_n$$.

We can then use Cramer’s rule to solve our equation. Cramer’s rule gives the solution to our system as a fraction of determinants. We use Lemma 15 to compute the multiplicative inverse of the denominator in additional space $3 r_n + O(\log r_n)$. Multiplying the inverse of the denominator with the numerator using their properties as well-endowed rings and writing into a (double) register used for the multiplicative inverse in additional space $O(\log r_n)$. If we then take the remainder in place in space $O(\log r_n)$, we can compute the solution to the system in $F_n$. $\square$

### An Overview of BCH Codes

The codes used to correct errors in our catalytic tape are so-called *Bose–Chaudhuri–Hocquenghem (BCH)* codes, as described by [16]. A BCH code has the following components:

1. An alphabet represented by a ‘small’ field *GF*(*q*).
2. Codeword length $n = q^m - 1$. Each position of the codeword is represented by a member of $F^*$ where $F^*$ is the multiplicative group of $F = GF(q^m)$ for a fixed value *m*. We may call $F = GF(q^m)$ the larger field.
3. Distance $$\delta $$.

And we make the following choices.

1. We set $q = p^{r_n}$ for a prime number *p* and $r_n$ that depends on the size of the input tape of the machine. Here *p* is fixed and we set it to $p = 2$ in this work.
2. $m = 1$, therefore the small field equals the large field $F = GF(q)$.
3. $\delta = 2e + 1$. It is well known that one needs a distance of at least $2e + 1$ to be able to correct *e* errors.

Together these choices form a $[p^{r_n} - 1, p^{r_n} - 1 - \delta , \delta ]$-code over an alphabet of size $p^{r_n}$. Ensuring that $p = 2$ means that we can interpret the catalytic tape as a sequence of elements in $GF(q) = GF(2^{r_n})$. Furthermore, we wish to have the property that by extending a word by a small amount we can turn any word into a codeword. We observe that codewords are defined as words that satisfy the following property for $i = 1, \dots , \delta - 1$.

1 $$\begin{aligned} s_i = \sum _{x \in F^*} d_x x^i = 0 \end{aligned}$$

Here the $d_x$ represents the value of the codeword stored at position *x*. Now presume we have a word of length *n* then we extend the word by adding entries, we call the list of added entries $C \subseteq F^*$. The added values can be set arbitrarily, therefore we obtain the following equations:

2 $$\begin{aligned} s_i = \sum _{x \in F^*} d_x x^i = \sum _{x \in F^* \setminus C} d_x x^i + \sum _{x \in C} d_x x^i = s_i' + \sum _{x \in C} d_x x^i = 0 \end{aligned}$$

for

3 $$\begin{aligned} - s_i' = \sum _{x \in \mathcal {F}^* \setminus C} d_x x^i \,. \end{aligned}$$

We observe that for every value of $i = 1, \dots , \delta - 1$, we obtain an equation. Each equation is linear in the $d_x$ for $x \in C$. These are the new data points we must calculate in order to turn an arbitrary word into a codeword. Overall this yields the encoding linear system with parameter $\delta - 1$ and $i = 1, \dots , \delta - 1$

4 $$\begin{aligned} \sum _{x \in C} d_x x^i = - s_i' \end{aligned}$$

In order to argue that a solution to this system always exists, we need the small field to equal the large field of the BCH code. This means $m = 1$. This is necessary because the value $s_i'$ lies in the large field of the code while the values of $d_x$ lie in the small field of the code. If these are the same, we can treat this as a linear algebra problem.

#### Lemma 17

By setting $|C| = 2e = \delta - 1$, adding this many members of the alphabet of a BCH code with $m = 1$, it is always possible to turn any string into a codeword.

#### Proof

As discussed, it is sufficient to show that Equation 4 always has a solution. In order to see this, observe that Equation 4 forms a linear system over the field *GF*(*q*) and that the matrix of this system is a Vandermonde matrix. Vandermonde matrices are always invertible. Thus a solution to this system always exists. $\square$

#### Corollary 18

Let *S* be a data string of *n* bits and $e \le \frac{1}{2} c /\log (c)$, then there exists a BCH code, with distance $\delta = 2e +1$ and codeword length $n + (2e + 1)\lceil \log (n + e) \rceil$.

#### Proof

We set $r_n = \lceil \log (n + e) \rceil$ therefore $q = 2^{\lceil \log (n + e) \rceil }$, therefore the alphabet of is of size $2^{\lceil \log (n + e) \rceil }$. We break the initial catalytic tape into blocks of length $\lceil \log (n + e) \rceil$, these blocks form the initial letters of the word. If $\lceil \log (n + e) \rceil \not \mid n$, we pad the last block of the catalytic tape with additional 0’s of free space, this requires at most $\lceil \log (n + e) \rceil - 1$ bits of free space. This gives a word consisting of $\lceil \frac{n}{(\lceil \log (n + e)\rceil )}\rceil$ letters. Now we use Lemma 17 and add 2*e* letters of size $\lceil \log (n + e) \rceil$, using $2e \lceil \log (n + e) \rceil$ of free space, such that these new members abide by Equation 4. This creates a codeword of length $n + (2e + 1)\lceil \log (n + e) \rceil$ as required. $\square$

#### Remark B.1

Note that *q* in this lemma is taken larger than strictly necessary. There are two requirements on *q*, namely *q* is the total number of letters we can use to construct a code-word, and that $\log (q)$ the number of bits of which one codeword exists. In this work we set $r_n$ to the number of bits required to represent one letter of the word, which gives the following equation for $r_n$:

$$\begin{aligned} 2^{r_n} \ge 2e + \frac{n}{r_n}, \end{aligned}$$

which our choice of $r_n$ satisfies, but might not always be optimal.

Given that this code exists and has the correct space complexity we will show that it can be space efficiently computed. Even before doing encoding and decoding, it is required to do an initialization step:

We now argue the initialization can be done space efficiently.

#### Lemma 19

Given a sequence of fields $F_n = GF(2{r_n})$, Algorithm 1 can be performed in space $3r_n + O(\log r_n)$.

#### Proof

We review each step of Algorithm 1 and review their space cost:

1. For step 1, use 14 to find an irreducible polynomial in space $$3 r_n + O(\log r_n)$$. Only $$r_n $$ is needed to store the result.
2. For step 2, we can pick and save the element of $GF(2)[\xi ]$ corresponding to $\xi$. This uses *O*(1) space if always done the same. This does not work for $r_n = 1$, but we can assume always $r_n > 2$.

$$\square $$

Encoding requires solving the linear equations given by Equation 4, finding the values $d_x$ for $x \in C$, the additional blocks that were appended. Solving these linear equations requires first calculating the quantities $s_i'$, given by Equation 3. We use the following algorithm to calculate a specific value $s_i$. By stopping prematurely, we can compute $s_i'$.

Now we give the space complexity of Algorithm 2.

#### Lemma 20

Given a sequence of positive integers $r_n$, the $s_i$ and $s_i'$ can be computed in space $6 r_n + 2 \lceil \log \delta \rceil + O(\log r_n)$, counting the $r_n$ space used to store the generator of the multiplicative group of $GF(2^{r_n})$ and an irreducible polynomial to represent $GF(2^{r_n})$. We assume that the generator is simple so does not use much space.

#### Proof

Every element of $GF(2^{r_n})$ uses space $r_n$. We use six of these. We also use two registers of size $\lceil \log \delta \rceil$. Multiplication and addition use overhead $O(\log r)$. $\square$

We present the BCH encoding algorithm, and argue that it is space efficient.

#### Lemma 21

It is possible to encode a word of length $2^{r_n} - \delta$ with an alphabet $F_n = GF(2^{r_n})$ and distance $\delta$ as a codeword of length $2^{r_n}$ with an additional space overhead of $O(e \log n) = (4e + 6) r_n + O(\log ^2 r_n)$, for $r_n = \log (e + c) \le \log (c) + 1$. Furthermore this encoding procedure is done in place. This implements the function $Enc_{BCH}$.

#### Proof

We look at the space complexity of every step of the encoding procedure.

1. Initialization costs $3r_n + O(\log r_n)$ space by Lemma 19.
2. For step 2, use Algorithm 2. This means we store $$2e r_n $$ values and use $$6r_n + 2\lceil \log (\delta )\rceil + O(\log r_n)$$ space.
3. For step 3, we use Lemma 16 which uses $(4e + 5) r_n + O(\log ^2 r_n)$ space.

Overall, this adds up to a space cost $(6e + 11) r_n +\lceil \log (\delta ) \rceil + O(\log ^2 r_n)$. Note also that encoding only blocks to the existing word, making this computation done in place. $\square$

That completes encoding. We now move to our analysis of decoding. We review the mathematics of the decoding.

We now describe the theory of the decoding algorithm. Decoding follows the procedure described in [16, 18] with some simplifications since we prioritize space over time. First, the syndrome $\textsf{syn}(p)$ of a message *p* is computed. The syndrome is defined as the collection of the $s_i$ values defined before. From the syndrome we compute the support of the error, $\textsf{supp}(p) = \{(x, p_x)_{x: p_x \ne 0}\}$ which is defined as the value of the error $p_x$ together with its position *x*. Then the error can be ‘subtracted’ from the word to give back the original codeword. The error correction method only works if the number of errors is at most $(\delta - 1)/2$ and hence we set $\delta = 2e + 1$. It is important for space efficiency that we store only the support of the error, instead of a full error string which would require too much space. The support on the other hand uses exactly $O(e \log c)$ space.

The decoding algorithm is a variation of Berlekamp’s BCH decoding algorithm. First, define the following polynomials using $M = \{x \in \mathcal {F}^* | p_x \ne 0\}$

5 $$\begin{aligned} \sigma (z)&= \prod _{x \in M} (1 - xz)&\omega (z)&= \sigma (z) \sum _{x \in M} \frac{p_x xz}{1 - xz} \end{aligned}$$

which both have degree at most $|M| \le (\delta - 1)/2$. Here $\sigma (z)$ is known as the error locator polynomial since the multiplicative inverses of its roots are the locations of the errors. Similarly, $\omega (z)$ is known as the evaluator polynomial since it gives the error since $\omega (x^{-1}) = p_x \prod _{y \in M, y \ne x} (1 - yx^{-1})$. Note that since these polynomials have no common zeroes, $\textrm{gcd}(\sigma (z), \omega (z)) = 1$.

It turns out that $\sigma (z)$ and $\omega (z)$ are the almost unique solutions to the congruence (with parameter $\delta - 1$)

6 $$\begin{aligned} S(z) \sigma (z) \equiv \omega (z) \pmod {z^\delta } \end{aligned}$$

where $S(z) = \sum _{l=1}^{\delta - 1} r_l z^l$. Suppose that $\sigma '(z),\omega '(z)$ are other solutions to this congruence then

7 $$\begin{aligned} \omega (z) \sigma '(z) \equiv \sigma (z) S(z) \sigma '(z) \equiv \sigma (z) \omega '(z) \pmod {z^\delta } \,. \end{aligned}$$

Therefore if we restrict the degree of both $\omega (z)$ and $\sigma (z)$ to be polynomials of degree at most $(\delta - 1) / 2$ then as polynomials it is also true that $\omega (z) \sigma '(z) = \sigma (z) \omega '(z)$ and therefore that $\omega (z) / \sigma (z) = \omega '(z) / \sigma '(z)$. So if we also require that $\omega (z)$ and $\sigma (z)$ are relatively prime and $\sigma (z)$ has constant coefficient 1, then $\omega (z), \sigma (z)$ are unique. We call the linear system over *GF*(*q*) from Equation 6 the decoding linear system with parameter $\delta$.

After setting the constant term of $\sigma (z)$ to be 1, the above congruence gives a linear system with $\delta$ unknowns and $\delta$ equations with coefficients in the field $GF(q^m)$. We use almost the same procedure as described in the encoding step and making use of Lemma 16 in order to solve this system. If less than $(\delta - 1) / 2$ errors are made, a solution is guaranteed to exist. However, we cannot force $\sigma (z)$ and $\omega (z)$ to be coprime in the linear system and as a result the solution may not be unique. Suppose $\sigma (z)$ and $\omega (z)$ have a common factor $\tau (z)$. Since $\omega (z)$ must have a constant coefficient 1, the constant coefficient of $\tau (z)$ must also be 1. But then $\tau (z)$ must have degree at least 1 and therefore $\sigma (z)$ and $\omega (z)$ both have degree at least 1 too high. Therefore, if more than one solution to the system exists, some of these solutions will have too high a degree. But we can test whether or not the system has more than one solution by evaluating the determinant of the matrix of that system. If the determinant is 0, we can repeat our procedure but now with $\delta$ replaced by $\delta - 2$ to get a smaller linear system. Repeating this procedure, either we find that the determinant is always 0, meaning that there are no errors to correct, or eventually that the determinant is nonzero and we can solve for polynomials $\sigma (z), \omega (z)$.

Having solved for polynomials $\sigma (z)$ and $\omega (z)$ we can iterate over all possible values of $z \in \mathcal {F}^*$ to find all roots to $\sigma (z)$ and then compute their inverses using a similar procedure to that described in the encoding step. The evaluation of this polynomial can be done space efficiently, similar to the evaluation of $s_i$ but much simpler in fact. Afterwards, we can evaluate $\omega (z)$ to compute the errors. This is not necessary when $q = 2$ and the error is guaranteed to be 1. Once these have been computed, storing the support of the error is space efficient and the catalytic tape can be corrected. This completes the decoding step. This procedure is performed by the following algorithm, and we give it space complexity.

#### Lemma 22

Algorithm 4 can be performed with space overhead $O(e \log n) = (6 + 4e) r_n + O(\log ^2 r_n)$, for $r_n = \log (e + c) \le \log (c) + 1$, including the cost of the initialization using Algorithm 1. Furthermore this decoding procedure is done in place. This implements the function $Dec_{BCH}$.

#### Proof

We review the cost of Algorithm 4 step-by-step. Steps that use a trivial amount of space are omitted.

1. Initialization costs $3r_n + O(\log r_n)$ space by Lemma 19.
2. By Lemma 20, the cost of Algorithm 2 is $5r_n + 2 \lceil \log \delta \rceil + O(\log s_i)$ not counting the space needed to store the irreducible polynomial.
3. Storing and computing a determinant using the method in the proof of Lemma 16 costs $2e r_n + O(\log ^2 r_n + \log e)$ space using access to an irreducible polynomial given in the initialization. The counter uses space $O(\log e)$.
4. Reuse space from step 3.
5. Solving the linear system by Lemma 16 uses space $4e r_n + 5r_n + O(\log ^2 r_n + \log e)$. We can reuse space used in step 3.
6. Evaluating a degree 2*e* polynomial can be done via Horner’s method. This uses one sum register, one double sized multiplication output register and one counter register. The multiplication output register has twice the size since before taking the remainder, the full product as a polynomial has to be stored. Since the irreducible polynomial has been precomputed, and we can compute remainders in place, we can evaluate a polynomial in additional space $$3r_n + \lceil \log 2e \rceil + O(\log r_n)$$. Iterating over all possible solutions uses an additional $$r_n $$ space. This procedure can recycle the space used in step 11. We use an additional register size $$\lceil \log 2e \rceil $$ to find the *i*th root. Counting the space used to store the irreducible polynomial means that this costs space $$(4e + 5)r_n + O(\log ^2 r_n + \log e)$$.
7. We add a (double-sized) multiplication output register for multiplication, a register to maintain the product, and another set of registers to iterate over all possible roots. Iterating over all roots not equal to $x^{-1}$ allows us to then compute $\alpha _{x_i}$. We then take the multiplicative inverse using Lemma 15. Overall, this uses space $(4e + 6) + O(\log r_n)$ by reusing registers.
8. Reusing the space from steps 11 and 12 we can compute the value of the error by multiplying $\omega (x^{-1})$ by $\alpha _{x_i}^{-1}$.

This covers all steps of Algorithm 4 with significant space costs. We ignore $O(\log \delta )$ space terms here, since these are all $O(\log n)$. This adds us to $(6 + 4e) r_n + O(\log ^2 r_n)$ space. Note that the correction of corresponding errors is done in place on the codeword. $\square$

## Footnotes

1 We cannot expect an unconditional separation between ${\textsf {CL}}$ and any ${\textsf {LCL}}$, as even separating ${\textsf {PSPACE}}$ from e.g. ${\textsf {TC}}^1(\subseteq {\textsf {CL}})$ remains wide open.

2 The main idea is that allowing virtually unlimited error in an exponentially small fraction of catalytic tapes gives us a strong form of the “compress-or-random” framework of previous papers; we can simulate a randomized algorithm using the catalytic tape as our source of randomness, and in the exponentially unlikely event the tape is not sufficiently entropic we simply erase it and run brute force. Formalizing this intuition and combining it with the results of [10, 24] gives a derandomization barrier to showing even ${\textsf {LCL}} [O(1)] = {\textsf {CL}}$ in this lossy setting, namely that randomized ${\textsf {TC}}^1$, which is not known to even be in $\P$, would reduce to the lossy code problem.

3 Technically because of our parameters, they can even be considered Reed-Solomon codes, which are a special case of BCH codes; nevertheless we follow the presentation of the more general code form.

4 Note that in principle the first *c* bits of $\tau _{enc}$ do not have to be equivalent to those of $\tau$. However due to $M_e$ being a lossy catalytic machine, it should still provide the same output when run on the first *c* bits of $\tau _{enc}$ as when run on $\tau$.

5 This construction is based on the solution to the so-called “almost impossible chessboard puzzle”; interested readers can find the setup and solution in videos on the YouTube channels 3Blue1Brown (https://www.youtube.com/watch?v=wTJI_WuZSwE) and Stand-up Maths (https://www.youtube.com/watch?v=as7Gkm7Y7h4). It can also be seen as the syndrome of the Hamming code.
